# Smokeless Tobacco Cessation in an Emergency Room in Rural West Virginia

**DOI:** 10.3389/fpubh.2022.811397

**Published:** 2022-04-06

**Authors:** Donald Reed, Kathy Danberry

**Affiliations:** ^1^Liberty University, Lynchburg, VA, United States; ^2^West Virginia State Department of Health and Human Resources, Charleston, WV, United States

**Keywords:** smokeless tobacco cessation, emergency room nursing, tobacco use, brief tobacco intervention, nursing-education

## Abstract

**Background:**

Emergency room nurses have a strong influence on the population of smokeless tobacco users. If healthcare providers address patient's tobacco use by using a brief intervention strategy (one minute or less), it increases the quit attempt rate threefold. The object of this study is to assess the effectiveness of asynchronous internet based brief tobacco intervention training with rural emergency room nurses.

**Methods:**

A 1-h asynchronous training session on smokeless tobacco use and the 2-A and 1-R (Ask, Advise, and Refer) brief tobacco intervention strategy were given to 13 emergency room nurses at a rural acute care hospital in West Virginia. Paired sample *t*-tests were used to compare the pre-and post-test results.

**Results:**

The 1-h training session produced significant and positive increases in all items measured: increased motivation to assist patients in quitting; increased knowledge of smokeless tobacco use, its dangers, and cessation processes; increased self-efficacy in implementing brief interventions; increased perception of tobacco cessation as important; increased perception of the effectiveness of tobacco cessation interventions; and increased acknowledgment of barriers and an awareness of how to deal with them.

**Conclusions:**

The results suggest that there is a significant potential benefit from training emergency room nurses. Brief tobacco interventions should be conducted by clinical staff during the medical history check, physical examination, or discharge phases of the emergency room visit.

## Introduction

Although the consequences of illness, disease, and death from tobacco use are well identified and known, to the extent that it is considered to be a chronic disease (1), it remains the single most preventable cause of disease and death in the United States (2). This is also the case in West Virginia, which continues to lead the nation in tobacco use, to an alarming extent. West Virginia has the highest state-specific prevalence of current adult use of cigarettes (26.7%), the second highest state-specific prevalence of current adult use of smokeless tobacco (8.5%), and the highest state-specific prevalence of any cigarette/smokeless tobacco use (i.e., any tobacco use) among U.S. adults in the nation (32.2%).

In a standardized review of over 14,000 emergency department admission screenings from across six states in the United States (including West Virginia), 47% of emergency room patients had used some form of tobacco within the past 30 days (3). This rate of tobacco use is more than twice the national average (4). A younger age, lower income, being male, and being a blue-collar worker have been found to be associated with the increased likelihood of tobacco use (5–7), which is viewed as a predictor of other substance abuse issues in emergency department patients (3, 8). Patients who link the cause of their emergency department visit to their tobacco use are more likely to attempt to quit and provide nursing staff with a teachable moment (9, 10). Motivation to quit tobacco use is the top variable in a quit attempt (11).

When patients in emergency departments are educated on the dangers of tobacco use and are referred to a treatment source, they are more likely to initiate contact with a treatment provider (12). Patients prefer a wide variety of tobacco cessation interventions that focus on individualized feedback and autonomy: nicotine replacement therapy options, referral to state quit lines and community cessation workshops, and inpatient cessation counseling (13).

Healthcare providers are more likely to conduct brief tobacco interventions that result in the client being referred to a national or state-level tobacco quit line (14). Quit line referrals, as a brief tobacco intervention, are more effective than self-help material alone (15). Cessation attempts increase when nurses are trained to implement brief tobacco interventions properly (16). Brief tobacco interventions result in increased quit attempts in low-income tobacco users, especially after they link their smoking to the cause of a pediatric emergency department visit: thus, linking the illness of a child to the adult tobacco use (17). Emergency department tobacco cessation interventions result in low three-month cessation rates, and thus interventions should be “simple, quick, and inexpensive” (18) if they are to be feasible.

Rural nurses are more likely to view tobacco cessation as part of their role than urban nurses (19). System-level cessation support is vital to interventions: pamphlets/self-help materials; a tobacco documentation system; and a policy that identifies tobacco use (19). Due to nurse's time constraints, a fax referral system to the state tobacco quit line will increase cessation attempts among tobacco users who present in the emergency department (15, 20).

### Research Question

With nursing staff often unable to attend in-person training, the study aims to assess the effectiveness of asynchronous internet based brief tobacco intervention training with rural emergency room nurses.

### Current State

Most nurses leave the subject of tobacco cessation to the provider. Only a few currently use any CDC-recommended intervention or refer smokeless tobacco users to the West Virginian tobacco quit line.

### Desired State

Emergency room nurses should feel that tobacco cessation is “very much” a part of their job duties. Nurses should typically spend 3–10 min with patients on tobacco cessation education, using CDC-recommended interventions. Furthermore, nurses should increase their knowledge and self-efficacy in the delivery of smokeless tobacco cessation interventions.

## Materials and Methods

This project was conducted in agreement with the West Virginian Division of Tobacco Prevention. The participants were 13 emergency room nurses at Welch Community Hospital in McDowell County, an acute care facility owned and managed by the West Virginian Department of Health and Human Resources and located in the rural coalfields of the state. The emergency room nurses included five licensed practical nurses, nine registered nurses, and one family nurse practitioner. Nurses from all work shifts participated–i.e., day shift, evening shift, and night shift. Participation was voluntary and participants acknowledge their own interest and willingness to be a part of the study, as shown by the informed consent. Nurses were recruited by a graduate student who also worked in the emergency room.

### Procedures

(1) All nurses participated in the asynchronous internet based brief tobacco intervention training on how to implement smokeless tobacco and the brief tobacco interventions (2-As & 1-R) for brief smokeless cessation counseling with emergency room nurses. Nurses will have the option to complete the same training via webinar if their schedule does not permit participation in the face-to-face training.
(2) Nurses used a guideline algorithm of the brief tobacco interventions, as provided by the CDC, with patients who use smokeless tobacco.
(3) When encountering tobacco users motivated to quit, nurses used fax quit line referrals or direct patient referrals (quit line cards) to the state tobacco quit line for proactive telephone counseling.
(4) Nurses received weekly support from a certified tobacco treatment specialist. This support was for nurses who have questions or encounter situations not covered in the training or the CDC algorithm.
(5) The intervention phase lasted 30 days at the site.

### Assessment Tools

Effective Training Assessment Instrument: Tobacco use status; motivation to help patients stop using tobacco; knowledge of performing tobacco interventions; confidence in ability to help patients quit; the importance of tobacco use in preventive care; perceived effectiveness of tobacco interventions; importance of addressing barriers; preparedness to provide intervention; how often the nurse sees patients affected by tobacco use; reactiveness in addressing tobacco use; success in helping patients quit; and how often the 2-As and 1-R brief intervention is used. The post-training instrument measured the same items (see Table 1). All items (with the exception of how often the 2-As and 1-R brief intervention is used) were assessed on a Likert-type, discrete analog scale from 0 to 10, with 0 being “not at all” and 10 being “the most ever.” The assessment tool used was created by Dr. Christine Sheffer from the University of Arkansas [see (21)].

**Table 1:** Means and standard deviations: focal variables.

**Variable**	**Mean**	**Std. Dev**.
Pretest: Motivated to help patients stop	8.31	1.65
Pretest: Knowledge of performing tobacco interventions	6.90	2.30
Pretest: Confidence in ability to help patients quit	6.25	2.22
Pretest: Importance of quitting tobacco in preventive care	8.54	1.71
Pretest: Effective tobacco interventions	5.77	1.69
Pretest: Importance of providing barriers	8.15	1.77
Pretest: Prepared to provide interventions	6.08	2.96
Pretest: How often the effects of tobacco on patients are witnessed	9.61	0.77
Pretest: Proactive in addressing tobacco usage	6.46	2.53
Pretest: Successful in helping patients quit	5.08	2.10
Pretest: How often 2-As and 1-R brief is used	4.31	1.03
Posttest: Motivated to help patients stop	9.30	1.33
Posttest: Knowledge of performing tobacco interventions	8.33	2.06
Posttest: Confidence in ability to help patients quit	8.50	1.78
Posttest: Importance of quitting tobacco in preventive care	9.50	0.85
Posttest: Effective tobacco interventions	8.00	2.30
Posttest: Importance of providing barriers	9.00	1.56
Posttest: Prepared to provide interventions	8.10	2.13
Posttest: How often the effects of tobacco on patients are witnessed	9.20	1.62
Posttest: Successful in helping patients quit	6.80	3.29
Posttest: How often 2-As and 1-R brief is used	2.30	1.33
Pretest: Percentage of correctly answered questions	80.77	15.79
Posttest: Percentage of correctly answered questions	96.15	5.46
*N = 13*		

Knowledge Assessment Instrument: The pre-training assessment instrument was a 20-item multiple choice questionnaire. The questionnaire tested nurse's knowledge of the following items: the difference in the level of harm between e-cigarettes and traditional cigarettes; levels of cravings with e-cigarettes; the effectiveness of nicotine patches with e-cigarettes; teen use of e-cigarettes; the role of the FDA oversight; the profile of an e-cigarette user; the use of an e-cigarette in tobacco cessation attempts; the level of nicotine in smokeless tobacco; prevalence data for West Virginia; the role of healthcare providers in tobacco cessation interventions; and the average number of cessation attempts before sustainable abstinence. The post-training instrument was measured by using the same items. Nurses had to achieve a score of at least 80% to pass the post-test and access the continuing education unit.

### Ethical Considerations

This study was approved by the Institutional Review Board at both Capella University and West Virginia University. Informed consent was received from all participants in the study. The identity of the participants remains anonymous and all data are reported in the aggregate format. An error was made in coding the pre- and post-effective training assessment instrument, as they were not blind coded correctly. Therefore, assessments were randomly assigned codes for the analysis.

## Results

For the analysis, the participants were grouped into one professional group: emergency room nurses (*n* = 13). All nurses identified as white (100%) in race. Most reported never having used tobacco regularly (46.2%), although almost one-quarter (23.1%) were current tobacco users and 7.7% were former tobacco users. The mean age of participants was 38.66 years, ranging between 24 and 57 years. The emergency room nurses included five licensed practical nurses, nine registered nurses, and one family nurse practitioner. Nurses from all work shifts participated. The mean number of years of practice as a nurse was 13.86 years.

To determine the relationship between the pre-test and post-test, a paired sample *t*-test was used. This was determined to be the appropriate method to use because two points of time were being compared (22).

*Confidence in ability to help patients quit* yields a statistically significant difference as a function of the independent variable (*t* = −2.910, *p* = 0.020) (see Table 2). The post-test score (M = 8.89) is higher than the pre-test score (M = 6.89) (see Table 2).

**Table 2:** Paired sample *t*-Test results: effective training is the key factor to increasing provider performance and proficiency in delivering brief tobacco interventions.

	**Pre-test**		**Post-test**			
**Variable**	**M**	**SD**	**M**	**SD**	***T***	***P-value***
Motivated to help patients stop	8.40	1.64	9.30	1.34	−1.274	0.235
Knowledge of performing tobacco interventions	7.71	1.89	8.57	1.81	−0.915	0.395
Confidence in ability to help patients quit	6.89	2.15	8.89	1.36	−2.910	0.020
Importance of quitting tobacco in preventive care	9.00	1.15	9.50	0.85	−0.958	0.363
Effective tobacco interventions	5.80	1.93	8.00	2.30	−1.779	0.109
Prepared to provide interventions	6.56	3.32	8.44	1.94	−1.350	0.214
How often the effects of tobacco on patients are witnessed	9.60	0.84	9.20	1.61	0.629	0.545
Proactive in addressing tobacco use	6.50	2.42	8.80	2.10	−2.725	0.023
Successful in helping patients quit	5.44	2.19	7.00	3.43	−1.036	0.330
How often 2-As and 1-R brief is used	4.60	0.70	2.30	1.34	5.438	0.000
Percentage of correct answers	80.77	15.79	96.15	5.46	−3.284	0.007

*df = 7; n = 10; all p-values are for two-tailed tests*.

*Proactive in addressing tobacco usage* yields a statistically significant difference as a function of the independent variable (*t* = −2.725, *p* = 0.023) (see Table 2). The post-test score (M = 8.80) is higher than the pre-test score (M = 6.50) (see Table 2).

*How often the 2-As & 1-R brief is used* yields a statistically significant difference as a function of the independent variable (*t* = 5.438, *p* = 0.000) (see Table 2). The pre-test score (M = 4.60) is higher than the post-test score (M = 2.30) (see Table 2). Note that the scale used was 1–5, with always = 1, usually = 2, about half the time = 3, seldom = 4, and never = 5. These data must be viewed as low numbers, meaning more frequency in using the brief intervention. Pre-and post-data reviews show a 100% increase in the frequency of using the brief intervention with patients.

*Percentage of correct answers* yields a statistically significant difference as a function of the independent variable (*t* =−3.284, *p* = 0.007) (see Table 2). The post-test score (M = 96.15) is higher than the pre-test score (M = 80.77) (see Table 2).

## Discussion

Emergency room nurses trained in smokeless tobacco knowledge and evidence-based brief tobacco interventions increase their knowledge of tobacco usage as well as develop positive attitudes toward tobacco intervention strategies such as the 2-As and 1-R method and the state tobacco quit line. These increases seem to suggest that training leads to higher rates of brief tobacco interventions with patients in the emergency room setting. The rates of cessation of the patients are not known.

With shrinking tobacco cessation budgets in West Virginia and across the nation, using asynchronous webinars as training platforms and focusing on healthcare providers to deliver brief tobacco interventions are cost-effective tools. In this case, almost 90% of the sample hospital's emergency room nurses were trained and willingly implemented the brief tobacco intervention, all at minimal cost. The same asynchronous webinar is now being used in healthcare facilities across West Virginia.

The project is sustainable without future funding. The webinar training and implementation by the nurses provide a skill set that need not be retaught, since the trained nurses can disseminate the knowledge they have acquired on how to conduct brief tobacco interventions to new nurses.

A limitation of this project is that it is based upon the self-reporting from emergency room nurses. As such, there is no direct evidence that long-term tobacco abstinence occurs. Further data collection and investigation are needed to provide evidence that the following occur as a result of the training provided: (a) increased motivation to assist patients in quitting; (b) increased knowledge of smokeless tobacco use, its dangers, and cessation processes; (c) increased self-efficacy in implementing brief interventions; (d) increased perception of tobacco cessation as important; (e) increased perception of the effectiveness of tobacco cessation interventions; and (f) increased recognition of the importance of barriers and preparedness to deal with the barriers.

The last limitation to note is that the participants were from one small acute care emergency room in West Virginia and not chosen at random. As such, generalizations from this data cannot be made due to the sample size.

While it is understood that this study can not be used to generalize its finding, this study does sustenance and parallel with the findings in a larger study conducted in 2009 by researchers at the University of Arkansas (21).

## Conclusion

The nurses at this rural hospital emergency room initiated the project with different levels of knowledge and different attitudes, motivations, self-efficacy, and practices toward smokeless tobacco cessation. The 1-h webinar training session produced significant and positive increases in all items measured: increased motivation to assist patients in quitting; increased knowledge of smokeless tobacco use, its dangers, and cessation processes; increased self-efficacy in implementing brief interventions; increased awareness of tobacco cessation as important; increased perception of the effectiveness of tobacco cessation interventions; and increased recognition of the importance of barriers and preparedness to deal with the barriers than before the training. Most importantly, pre-and post-data reviews show a 100% increase in the frequency of using the brief intervention with patients.

The results suggest large potential to benefit from training emergency room nurses. In the literature, these brief tobacco interventions should be conducted by clinical staff during the medical history check, physical examination, or discharge phases of the emergency room visit (12). The 2-As and 1-R brief tobacco intervention can take <3 min to complete, and is effective at helping tobacco users increase their quit attempt (23). Tobacco users whose visit to the emergency room can be linked to their tobacco use are more likely to have a quit attempt after the visit (9, 10, 24, 25).

## Data Availability Statement

The original contributions presented in the study are included in the article/supplementary material, further inquiries can be directed to the corresponding authors.

## Ethics Statement

The studies involving human participants were reviewed and approved by Capella University and West Virginia University. Written informed consent for participation was not required for this study in accordance with the national legislation and the institutional requirements.

## Author Contributions

DR is the lead researcher and editor. KD was an assistant editor on this project and also part of the funding agency. Both authors contributed to the article and approved the submitted version.

## Funding

This project was funded by the West Virginia Division of Tobacco Prevention.

## Conflict of Interest

The authors declare that the research was conducted in the absence of any commercial or financial relationships that could be construed as a potential conflict of interest.

## Publisher's Note

All claims expressed in this article are solely those of the authors and do not necessarily represent those of their affiliated organizations, or those of the publisher, the editors and the reviewers. Any product that may be evaluated in this article, or claim that may be made by its manufacturer, is not guaranteed or endorsed by the publisher.
